# Early Prediction of Diabetes Using an Ensemble of Machine Learning Models

**DOI:** 10.3390/ijerph191912378

**Published:** 2022-09-28

**Authors:** Aishwariya Dutta, Md. Kamrul Hasan, Mohiuddin Ahmad, Md. Abdul Awal, Md. Akhtarul Islam, Mehedi Masud, Hossam Meshref

**Affiliations:** 1 Department of Biomedical Engineering (BME), Khulna University of Engineering & Technology (KUET), Khulna 9203, Bangladesh; 2 Department of Biomedical Engineering (BME), Military Institute of Science and Technology (MIST), Mirpur Cantonment, Dhaka 1216, Bangladesh; 3 Department of Electrical and Electronic Engineering (EEE), Khulna University of Engineering & Technology (KUET), Khulna 9203, Bangladesh; 4 School of Information Technology and Electrical Engineering, The University of Queensland, Brisbane, QLD 4072, Australia; 5 Electronics and Communication Engineering (ECE) Discipline, Khulna University (KU), Khulna 9208, Bangladesh; 6 Statistics Discipline, Khulna University (KU), Khulna 9208, Bangladesh; 7 Department of Computer Science, College of Computers and Information Technology, Taif University, P.O. Box 11099, Taif 21944, Saudi Arabia

**Keywords:** artificial intelligence, diabetes prediction, ensemble ML classifier, filling missing value, outlier rejection, South Asian diabetes dataset

## Abstract

Diabetes is one of the most rapidly spreading diseases in the world, resulting in an array of significant complications, including cardiovascular disease, kidney failure, diabetic retinopathy, and neuropathy, among others, which contribute to an increase in morbidity and mortality rate. If diabetes is diagnosed at an early stage, its severity and underlying risk factors can be significantly reduced. However, there is a shortage of labeled data and the occurrence of outliers or data missingness in clinical datasets that are reliable and effective for diabetes prediction, making it a challenging endeavor. Therefore, we introduce a newly labeled diabetes dataset from a South Asian nation (Bangladesh). In addition, we suggest an automated classification pipeline that includes a weighted ensemble of machine learning (ML) classifiers: Naive Bayes (NB), Random Forest (RF), Decision Tree (DT), XGBoost (XGB), and LightGBM (LGB). Grid search hyperparameter optimization is employed to tune the critical hyperparameters of these ML models. Furthermore, missing value imputation, feature selection, and K-fold cross-validation are included in the framework design. A statistical analysis of variance (ANOVA) test reveals that the performance of diabetes prediction significantly improves when the proposed weighted ensemble (DT + RF + XGB + LGB) is executed with the introduced preprocessing, with the highest accuracy of 0.735 and an area under the ROC curve (AUC) of 0.832. In conjunction with the suggested ensemble model, our statistical imputation and RF-based feature selection techniques produced the best results for early diabetes prediction. Moreover, the presented new dataset will contribute to developing and implementing robust ML models for diabetes prediction utilizing population-level data.

## 1. Introduction

Diabetes is an illness that is becoming increasingly severe and morbid in both industrialized and developing countries [1]. When pancreas cells cannot produce enough insulin, blood sugar levels rise, which can negatively impact a number of organs, most notably the eyes, kidneys, heart, and nerves [2]. According to Fitzmaurice et al. [3], the percentage of adults around the world who had diabetes in 2017 was roughly 8.8%, and it is projected that by 2045, this percentage will rise to 9.9%. A recent study sheds light on the seriousness of diabetes by revealing that the condition affects half a billion individuals worldwide and that this figure is expected to increase by 25.0% and 51.0% by the years 2030 and 2045, respectively [4]. It is estimated that around 1.5 million individuals died directly from diabetes in the year 2012, while 2.2 million perished from cardiovascular diseases, chronic kidney disease, and tuberculosis [5]. The percentage of diabetes patients in the intended geographic region, which is Bangladesh, skyrocketed to 10.0% in 2011, up from 4.0% in 1995–2000, 5.0% in 2001–2005, and 6.0% in 2006–2010, in consonance with Akter et al. [6]. According to Danaei et al. [7], diabetes may be broken down into three primary subtypes, namely type I diabetes (also known as juvenile diabetes), type II diabetes, and type III diabetes (also referred to as gestational diabetes). An idiopathic issue causes type I diabetes [8]. It accounts for around 5.0% to 10.0% of all cases of diabetes [9,10] and is generally diagnosed in children and young adults [11]. Type II diabetes is characterized by inadequate production of insulin by the pancreas. It accounts for more than 90.0% of all instances of diabetes according to Shi and Hu [12], and is not only prevalent in those older than 45 years old but also in younger age groups such as children, adolescents, and young adults. Gestational diabetes is diagnosed in expectant mothers who have never been diagnosed with diabetes but who develop hyperglycemia during pregnancy. Approximately 2.0% to 10.0% of all pregnant women are affected by gestational diabetes, which can become worse or go away after birth [13]. It is possible to manage diabetes and keep it under control if an accurate early diagnosis is made; however, there is no cure for diabetes in the long run. Due to non-linearity, non-normality, and the complicated and linked structure in the majority of medical data, diabetes data categorization is a challenging endeavor [14]. Additionally, the presence of a large number of outliers in the dataset, in addition to missing or null values, affects the outputs of the diabetes classification [15].

Different machine learning (ML) algorithms, for instance, Linear Discriminant Analysis (LDA) [16], Quadratic Discriminant Analysis (QDA) [17], Naive Bayes (NB) [18], Support Vector Machine (SVM) [19], Artificial Neural Network (ANN) [20], Decision Tree (DT) [21], J48 [22], Random Forest (RF) [23], Logistic Regression (LR) [24], AdaBoost (AB) [25], and K-nearest Neighborhood (KNN) [26] have been employed in the prediction of diabetes diseases [14,27,28]. Researchers in [29] worked on different crucial features and the RF algorithm to forecast diabetes. The authors in [30] used three distinct ML classifiers: NB, DT, and SVM, in order to predict diabetes and found that NB provided the highest AUC value. A group of researchers in [31] applied various ML classifiers, such as KNN, DT, RF, AB, NB, and XGBoost (XGB). They have proposed a weighted ensemble ML model with the highest possible AUC value in recent studies. The authors in [32] recommended an ML-based diabetes prognosis system by applying the DT algorithm. Their primary concern was to identify diabetes at the candidates’ specific age. Moreover, in [33], the authors have suggested a predictive model for classifying diabetes based on several criteria employing the CART and Scalable RF. They reached the conclusion that the scalable RF model was more accurate than the standard RF model used in this predictive model. In [34], the ensemble AB model performed better than the Bagging ensemble model when it came to classifying diabetes mellitus (DM). This was determined by analyzing and applying the AB and Bagging ensemble methods and employing J48 (c4.5)-DT. The authors of [35] built a prediction model with two sub-modules: ANN and Fasting Blood Sugar (FBS). Following that, the DT algorithm was applied in order to identify the symptoms of diabetic patients correctly. In a similar vein, researchers working on [36] have utilized a variety of ML techniques, including SVM, AB, Bagging, KNN, and RF algorithms. Table 1 delineates several ML-based pipelines for diabetes classification employed in the previous literature with their respective datasets, missing data imputation techniques, feature selection methods, the number of features selected in that study, classifier, and their results in various evaluation metrics.

Even though numerous ML-based strategies have already been published in many research articles, the advancement in diabetes prognosis in recent years is still in the impoverished phase because of the paucity of efficacious and robust models. Determining a patient’s risk and susceptibility to a persistent condition such as diabetes is challenging. Early detection of diabetes lowers medical expenses and the possibility of developing more severe health issues. It is crucial that inferences may be drawn with accuracy from instantly observable medical signs, even in crises where a patient may be unconscious or unable to communicate, to assist doctors in making more effective choices for patient treatment in high-risk circumstances. Typically, the early signs of diabetes are very subtle. Therefore, ML-based advancements make early diabetes identification and diagnosis by automated procedure more likely and effective than the traditional approach of manually identifying diabetes, such as measuring blood glucose directly. The advantages include reduced burden for medical professionals and a lower likelihood of human error. We are attempting to apply a method that does not involve invasive procedures and uses ML approaches to forecast the early phases of a diabetic patient. This will allow the patient to be more cautious about their lifestyle to avoid potential complications. In the case of an intrusive procedure in which a blood glucose test is required, we would be able to make an early forecast in advance of the event taking place. Besides this, it reduces the hassle of going to the pharmacy to buy glucose strips and check the glucose level on time, which intensively reduces medical expenses as well as time.

The current research paper covers the following essential contributions:

- Introducing a new Diabetes Diseases Classification (DDC) dataset from the northeastern part of South Asia (Bangladesh).
- Recommending a DDC pipeline by proposing a weighted ensemble classifier using various ML frameworks for classifying this DDC dataset.
- Fine-tuning the hyperparameters of various ML-based models using the grid search optimization approach.
- Incorporating extensive preprocessing in the DDC pipeline, which comprises outlier rejection, missing value imputation, and feature selection techniques.
- Conducting extensive research for comprehensive ablation studies using various combinations of ML models to achieve the best ensemble classifier model, incorporating the best preprocessing from previous experiments.

The remainder of the article is structured as follows: Section 2 represents the proposed DDC dataset and ensemble ML models with different preprocessing in the introduced DDC pipeline. In Section 3, various extensive experimental results are presented with proper explanations and ablation studies. Finally, Section 4 concludes the article by abstracting future work directions with prospective applications.

## 2. Materials and Methods

This section describes the materials and methods employed in this experiment. Section 2.1, Section 2.2 and Section 2.3 describe our proposed datasets, framework, and evaluation criteria, respectively.

### 2.1. Proposed Datasets

When the proportion of one class is higher than the other, there is an imbalanced distribution of classes in the datasets. Classes with a substantial number of instances are referred to as majority classes, whereas classes with fewer instances are known as minority classes [46]. Our newly introduced DDC-2011 dataset has 4751 diabetes cases and 2814 non-diabetic cases. Similarly, the DDC-2017 dataset has a total of 3492 and 4073 diabetes and non-diabetic classes, respectively. Moreover, there are no prediabetes cases in the datasets (see details in Table 2). Therefore, this is a binary classification problem. A class imbalance problem emerges when the frequency of one class (for example, cancer) can be 1000 times lower than that of another class (for example, healthy patient) [47]. The majority class samples outnumber the minority class samples according to the class ratios, which can be 100 to 1 or 1000 to 1 or so on [48]. However, in our proposed datasets, the imbalance between majority and minority classes is significantly low (see details in Table 2), considering this a class imbalance problem. Therefore, DDC datasets are standard datasets [49], with an approximately equal number of samples in each class. Consequently, this article does not have to deal with the data imbalance problem.

#### 2.1.1. Data Source

This study was conducted utilizing Bangladesh Demographic and Health Survey (BDHS (https://dhsprogram.com/, accessed on 20 September 2022)) datasets in 2011 and 2017–2018 (see details in Table 3). The BDHS records data nationally on people’s socioeconomic characteristics, demographics, and numerous health factors. Two-stage stratified cluster sampling has been employed to accumulate data from selected households and surveyed through face-to-face interviews by the trained staff(s). We utilized totals of 5223 respondent information aged 35 years and above who tested blood pressure and glucose level in BDHS-2011. Furthermore, 12,119 respondents aged 18 years and above were used in the 2017–2018 BDHS survey. We consolidated the two BDHS datasets to create a substantially large sample to specify the risk factors for DM accurately.

#### 2.1.2. Study Variables

A biomarker questionnaire was provided by the BDHS program to collect information regarding HTN and DM diagnosis and treatments. Following the World Health Organization (WHO) recommended measurement, these surveys generally gathered records of plasma glucose levels. Trained health technicians recorded DM data through HemoCue Glucose 201 Analyzer. To quantify blood glucose levels, BDHS applied WHO cut-off levels. The fasting blood glucose level was ≥7.0 mmol/L, indicating the existence of DM and categorized as “Yes”. Here, prediabetes (PBG: 6.0–6.9 mmol/L with no medical care) and diabetes-free (PBG: <6.0 mmol/L) varieties were incorporated according to the BDHS classification procedure and categorized as “No”. However, the different categorical and continuous independent variables are represented in Table 3. The covariates used in the study are the age of the respondent (continuous), sex (male or female), educational level (no formal education, up to the primary, up to secondary, up to higher secondary), economic status (poorer, poor, middle, rich, richer), body mass index (continuous), occupation type (factory workers, beggars, boatmen, domestic servants, construction workers, brick breakers, road builders, rickshaw drivers, poultry raisers, cattle raisers, fishers, farmers, and agricultural workers, retired person, religious leader, housewife, businessman, family welfare visitor, teacher, accountant, lawyer, dentist, nurse, doctor, tailor, carpenter, unemployed/student, and landowner), eating habit (specified, anything), drinking coffee (no or yes), place of residence (urban or rural), division (Barisal, Chittagong, Dhaka, Khulna, Rajshahi, Rangpur, Sylhet, Mymensingh), average of diastolic (continuous), and the average of systolic (continuous).

### 2.2. Proposed Methodologies

**Figure 1:**
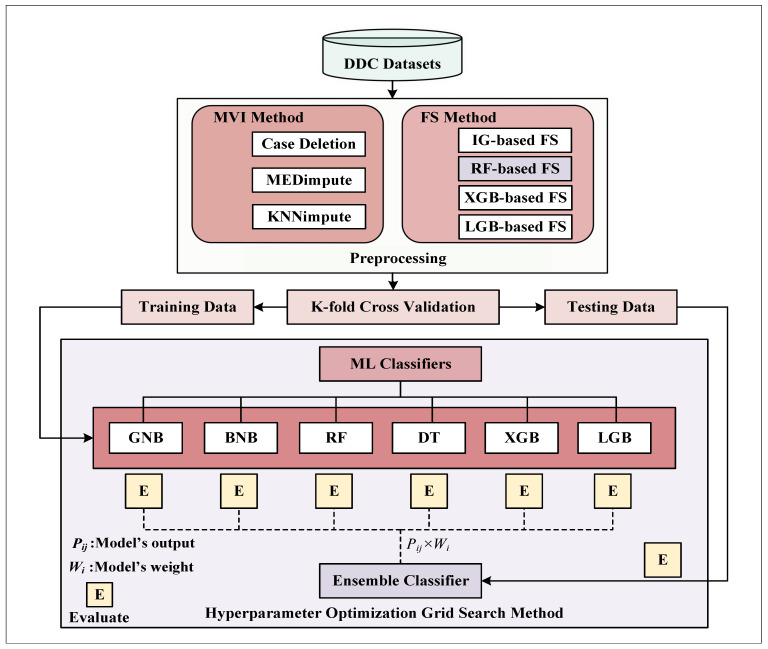
Block diagram of the proposed workflow incorporating various ML-based classifiers, a pre-processing step, and hyperparameter tuning through grid search optimization.

The overall workflow of this article has been illustrated in Figure 1 and essentially incorporates and investigates a preprocessing method and an ensemble ML classifier with hyperparameter optimization [50], Missing Value Imputation (MVI), and Feature Selection (FS) schemes are included in the suggested preprocessing. Additionally, K-fold cross-validation is applied to validate the proposed system’s robustness by analyzing the inter-fold variations. However, the different integral parts of our recommended DDC system are briefly explained in the following subsections.

#### 2.2.1. Missing Value Imputation (MVI)

A trainable automated classification decision-making framework entirely relies on a dataset. However, the practical dataset commonly includes an abnormal proportion of missing values, typically represented as NaNs, null, blanks, undefined, or similar place-holders [15]. Therefore, missing values in a dataset must be eliminated or imputed to develop a generic, robust, and effective classification model. Unlike the case deletion strategy, numerous statistical and ML approaches are employed extensively to handle data missingness in an incomplete dataset. For MVI purposes, median and KNN-based imputation techniques have been applied most frequently for several decades [15,51]. Thus, this article integrates median-based statistical and KNN-based ML imputation approaches and a case deletion strategy, which is portrayed in Figure 1. Moreover, Algorithm 1 illustrates the procedures used in the latter two MVIs.
**Algorithm 1:** The procedure for applying the MVI method
  **Input**: An uncurated column vector with n-samples ($X_{in}={\left[x_{1},\_,x_{3},\ldots ,x_{n}\right]}^{T}$),      where $x_{i}\in R$
  **Result**: A curated column vector with n-samples ($X_{out}={\left[x_{1},x_{2},x_{3},\ldots ,x_{n}\right]}^{T}$),      where $x_{i}\in R$
_1_  Impute the missing values using the following equation
$$\;\;\;\;\;\;\;X_{out}\left(s_{i}\right)=\left\{\begin{array}{cc} E_{j}, & \mathrm{for}\;i\mathrm{th}\;\mathrm{missing}\;\mathrm{sample}\;\mathrm{in}\;X_{in}\\ x_{i}, & \mathrm{for}\;\mathrm{other}\;\mathrm{cases} \end{array}\right.$$
  where $s_{i}$ is the *i*th sample of $X_{out}$ and $E_{j}$ is the estimated or predicted value in *i*th   position for *j*th attribute


#### 2.2.2. Feature Selection (FS)

FS is a fundamental strategy for determining which features are most likely acceptable for a specific ML model. FS approaches are commonly implemented in model simplification for more straightforward interpretation, reduced training times, reduced dimensionality, enhanced predictive accuracy by choosing the relevant features, and avoiding over-fitting [52,53]. Among the supervised, semi-supervised, and unsupervised FS procedures, the supervised FS method typically outperforms the others [31,54]. Therefore, for executing the ablation analyses for our suggested DDC datasets, this paper employs the four most typically exploited supervised FS techniques: RF, Information Gain (IG) [55], XGB [56], and LightGBM (LGB) [57], to minimize attribute redundancy. These four FS approaches are discussed shortly in the subsequent paragraphs.

##### RF-Based FS

RF is a tree-based method and is applied as an FS technique. It simply ranks the features based on how successfully it enhances the purity of the node, minimizing all trees’ impurities. The nodes consisting of the most significant impurity reduction appear at the onset of the trees, whereas a slight reduction in nodes’ impurity appears especially towards the tree’s end. As a result, a subset of the relevant features can be obtained by trimming the trees below a particular node. In Algorithm 2, the stages for the RF-based FS are described.
** Algorithm 2:** The procedure for applying RF-based FS method
   **Input**: The d-dimensional data, $X_{in}\in R^{n\times d}$ and result, Y∈[0,1]
   **Result**: The reduced m-dimensional data, $X_{out}\in R^{n\times m}$, where m < d
   _1_ Calculate a tree’s Out of Bag (OOB) error.
   _2_ When primary node *i* is separated in $X_{in}$, allocate per adherence with ${\tilde{P}}_{i}$ to minor    nodes at random, where the comparative frequency of occurrences is ${\tilde{P}}_{i}$, that    previously followed the tree in the same direction.
   _3_ Recalculate tree’s OOB error (follow step 2).
   _4_ Determine the contrast in OOB errors between the initial and recalculated errors.
   _5_ Reapply previous steps (1 to 4) for each tree, the total importance score (F) is then    calculated employing the average deviation across all trees.
   _6_ Choose the high scores (F) of top-m features as well as preserve them in $X_{out}$.


##### IG-Based FS

In ML, IG is an entropy-based feature selection strategy described as the vast information provided by the text category’s feature elements. In order to examine the significance of lexical items for classification, IG is calculated by determining how much of a term can be used for the information classification. The mathematical expression of IG is exhibited in Equation (1).    

(1) $$\begin{array}{c} G\left(D,t\right)=-\sum_{n=1}^{m}P\left(C_{i}\right)logP\left(C_{i}\right)+P\left(t\right)P\left(C_{i}|t\right)logP\left(C_{i}|t\right)+P\left(t\right)P\left(C_{i}|\bar{t}\right)logP\left(C_{i}|\bar{t}\right) \end{array}$$

where *C* is a set of collections of documents in which feature *t* does not exist. The value of G(D,t) is greater if feature *t* is selected. If a maximum value of G(D,t) is desired, the values of P(t) and $P(\bar{t})$ should be lower. Algorithm 3 depicts the procedures used for the IG-based FS.
**Algorithm 3:** The procedure for applying the IG-based FS method
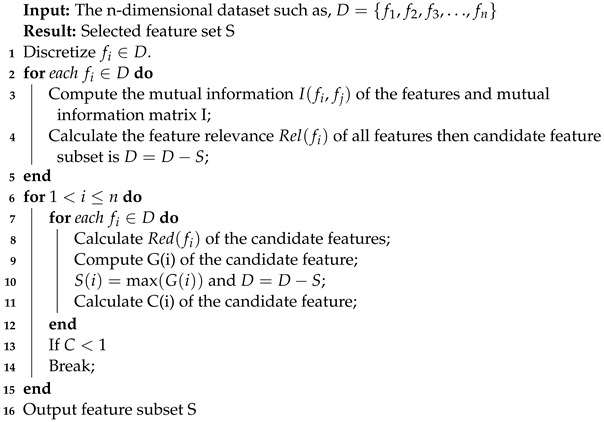


##### XGB- and LGB-Based FS

XGB and LGB are the executions of gradient boosting-based feature selection methods, ensemble strategies that use regularized learning, and the block structure of cache-aware tree-based learning. The gain score per tree partition results from these models, and the average growth is utilized to calculate the conclusive feature’s stature value. Eventually, the top-m indexed features are selected depending on the gain, as explained in Algorithms 4 and 5.
**Algorithm 4:** The procedure for applying the XGB diabetes detection model
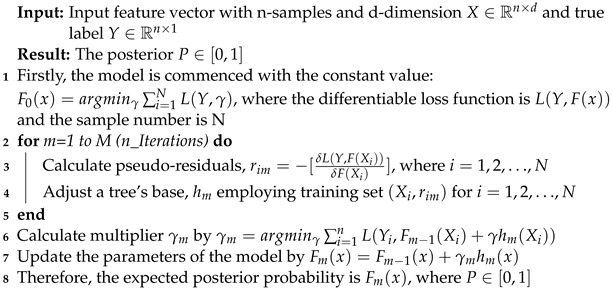


**Algorithm 5:** The procedure for applying the LGB diabetes detection model

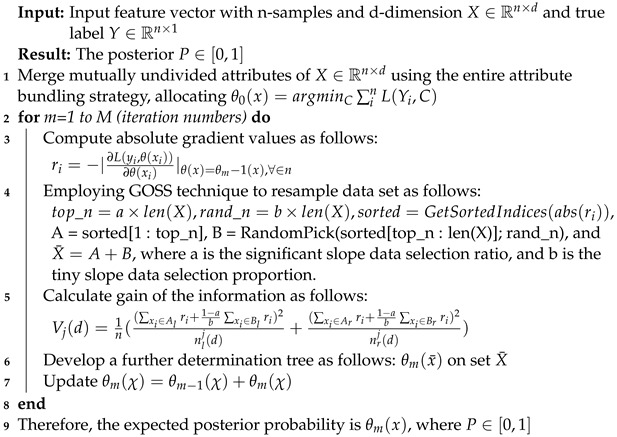



#### 2.2.3. K-Fold Cross-Validation

K-fold Cross-Validation (KCV) is one of the most extensively employed methods for selecting classifiers and predicting error [58]. The DDC datasets were divided into K numbers of the folds, training the models using the K-1 folds. Then we fine-tuned the hyperparameters by applying the grid search algorithm [59]. The best hyperparameters and unrevealed testing data were exploited to assess the models’ performance in the outermost loop (K times). Additionally, the stratified KCV has been implemented to restore each class’s constant percentage of samples because the DDC dataset includes both positive and negative samples. The final evaluation metrics were computed by employing Equation (2) [31].

(2) $$\begin{array}{c} M=\frac{1}{K}\times \sum_{n=1}^{K}P_{n}\pm \sqrt{\frac{\sum_{n=1}^{K}{\left(P_{n}-\bar{P}\right)}^{2}}{K-1}} \end{array}$$

where *M* is the final performance metric for the classifiers, *K* represents fold numbers, and $P_{n}\in R$.

#### 2.2.4. Hyperparameter Optimization

Since ML algorithms are sensitive to multiple hyperparameters, they need the best batch of hyperparameters [31,60,61]. However, grid search is one of the most fundamental approaches, defining a set of finite numbers per hyperparameter and analyzing the Cartesian product of these sets [61]. Let Ω to be the problem parameters space $P=(p_{1},p_{2},\ldots ,p_{m})$ across which the p-value should be maximized. A grid search strategy can be easily set up for each element of *P* by constructing a lower and upper vector limits such as $L=(l_{1},l_{2},\ldots ,l_{m})$ and $U=(u_{1},u_{2},\ldots ,u_{m})$, where *n* numbers of uniformly spaced points. Eventually, the highest of these values is elected once each pair of points has been computed. Six different kinds of ML optimized algorithms’ hyperparameters are summarized in Section 3.3.

#### 2.2.5. ML Classifiers

In this article, various ML classification algorithms such as GNB, BNB, DT, RF, XGB, and LGB are trained and evaluated for diabetes detection. The algorithmic processes of these ML models are explained in the following paragraphs.

##### GNB and BNB Classifier

The Bayesian approaches such as GNB and BNB are supervised learning-based algorithms. These algorithms are established on the principle of the Bayesian theorem and the presumption of conditional freedom between all the features, which provide the class variable’s value (see Algorithm 6). GNB employs a Gaussian operation as a likelihood of the features, whereas BNB utilizes multivariate Bernoulli distributions.
**Algorithm 6:** The procedure for applying the GNB and BNB diabetes detection model
   **Input**: Input feature vector with n-samples and d-dimension $X\in R^{n\times d}$ and true      label $Y\in R^{n\times 1}$
   **Result**: The posterior P∈[0,1]
 _1_ Calculate the prior as $P\left(Y=C_{j}\right)=\frac{n_{j}}{n},\forall_{j}\in C$, and $n_{j}$ is the sample in $j^{t}h$ class.
 _2_ Determine the posterior probability of the output as follows:    $P\left(C_{j}|X\right)=\frac{P\left(X|C_{j}\right)\times P\left(Y=C_{j}\right)}{P(X)}$, which $P(X|C_{i})$ is the predictor’s likelihood for a given    class $\left(\forall_{j}\in C\right)$.


##### RF Classifier

The RF classifier applies the bagging strategy to the individual trees present in the ensemble, as described in Algorithm 7. The training sample is then substituted with a random sample, and trees are fitted to these samples. The number of trees in the ensemble is a variable that can be learned spontaneously utilizing out-of-bag errors.
**Algorithm 7:** The procedure for applying the RF diabetes detection model
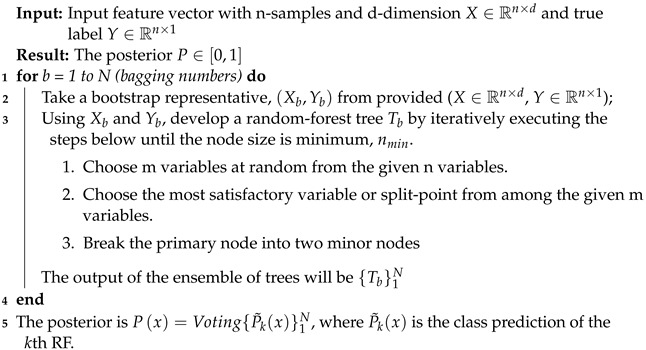


##### DT Classifier

DT adopts a tree structure to develop classification models (see Algorithm 8), splitting a dataset into progressively smaller subgroups. Decision nodes with at least two branches and leaf nodes indicating a classification or decision are the outcomes in a tree. Furthermore, the root node is the highest decision node in a tree that approximates the best prediction.
**Algorithm 8:** The procedure for applying the DT diabetes detection model
    **Input**: Input feature vector with n-samples and d-dimension $X\in R^{n\times d}$ and true     label $Y\in R^{n\times 1}$
    **Result**: The posterior P∈[0,1]
   
  _1_ Divide $\theta =(j,t_{m})$ into $Q_{left}\left(\theta \right)$ and $Q_{right}\left(\theta \right)$ subsets, where θ consisting of a feature, j and threshold, $t_{m}$.
   
  _2_ Use an impurity function (H), which are given below, to calculate the impurity at the $k^{th}$
    node, $G\left(Q,\theta \right)=\frac{n_{left}}{N_{m}}H\left(Q_{left}\left(\theta \right)\right)+\frac{n_{right}}{N_{m}}H\left(Q_{right}\left(\theta \right)\right)$, where     $H=\sum_{c}P_{mC}\times \left(1-P_{mC}\right)$ or $H=-\sum_{c}P_{mC}\times log\left(P_{mC}\right)$ and     $P_{mC}=\frac{1}{N_{m}}\sum_{x_{i}\in R_{m}}I\left(y_{i}=C\right)$
   
  _3_ Reduce the impurity by selecting the parameters, $\theta^{*}=argmin_{\theta }G\left(Q,\theta \right)$.
   
  _4_ Reapply the preceding steps for subsets $Q_{left}\left(\theta^{*}\right)$ and $Q_{right}\left(\theta^{*}\right)$ until depth reach to     $N_{m}<samples\;\left(minimum\right)$ or $N_{m}=1$.


##### XGB Classifier

XGB classifier is a boosting strategy in an ensemble model that consists of various models to increase prediction accuracy. Subsequent models correct the errors generated by prior models by applying weights to the models in this boosting method (see Algorithm 4).

##### LGB Classifier

LGB is based on DT techniques, employing a technique known as Gradient-based One-side Sampling (GOSS) and Exclusive Feature Bundling (EFB), which takes advantage of leaf-and level-wises tactics to speed up the training process [62,63] (see Algorithm 5).

##### Proposed Ensemble Classifier

The ensemble of the ML model is a prevalent technique for increasing performance by combining a group of classifiers [31,64,65]. Integrating the outputs from different classifier models in ensemble procedures can boost diabetes prediction accuracy. The six different ML models, as previously explained (GNB, BNB, RF, DT, XGB, LGB), are utilized for the ensemble frameworks as they can enhance the effectiveness of ML-based classifiers [31,66] and outperform in numerous medical fields, for instance, pneumonia, diabetic retinopathy, and measles vaccination uptake classifications [64,67,68]. We caluculate each models’ output, $Y_{j}$, $\left(j=1,2,3,\ldots ,m=6\right)\in R^{C}$ considering C=2 (whether diabetes patient, $C_{1}$ or not $C_{2}$) and confidence values $P_{i}\in R\;\left(i=1,2\right)$ on the unrevealed test data where $P_{i}\in \left[0,1\right]$ and $\sum_{i=1}^{C}P_{i}=1$. In this paper, Equation in (3) has been leveraged to achieve weighted aggregation of multiple ML algorithms.

(3) $$P_{i}^{en}=\frac{\sum_{j=1}^{m=6}\left(W_{i}\times P_{ij}\right)}{\sum_{i=1}^{C=2}\sum_{j=1}^{m=6}\left(W_{i}\times P_{ij}\right)}$$

where $W_{j}$ is the weight of corresponding *j*th classifiers’ AUC. The ensemble model’s output, $Y\in R^{C}$ contains the confidence values $P_{i}^{en}\in \left[0,1\right]$. The ultimate class label of our proposed DDC datasets’ unseen test data, X∈R from the ensemble framework will be $C_{i}$ if $P_{i}^{en}=max\left(Y\left(X\right)\right)$.

### 2.3. Evaluation Metrics

In this study, different types of performance metrics were utilized. This is related to why an ML model may perform well with one measurement from one evaluation metric while performing poorly with the other measurement from another. In order to ensure that an ML model is operating appropriately and optimally, various evaluation metrics must be employed. This article’s extensive experiments are evaluated by using a variety of metrics, including sensitivity (Sn), specificity (Sp), accuracy (Acc), and the receiver operating characteristic (ROC) curve with AUC value [15,69,70], which are estimated in this way:

(4) $$\begin{array}{c} Sn=\frac{TP}{TP+FN} \end{array}$$

(5) $$\begin{array}{c} Sp=\frac{TN}{TN+FP} \end{array}$$

(6) $$\begin{array}{c} Acc=\frac{TP+TN}{TP+FP+TN+FN} \end{array}$$

where TP, FN, TN, and FP indicate the numbers of true positives, false negatives, true negatives, and false positives, respectively. The Sn and Sp are applied to estimate type II errors (patient who has diabetes but incorrectly recognized as a non-diabetic patient) and type I errors (patient who is non-diabetic but incorrectly recognized as a diabetic patient), which are calculated by utilizing Equations (4) and (5), respectively. On the other hand, Acc calculates the total accurately identified samples among all samples present in the datasets using Equation (6). Additionally, the ROC curve demonstrates the classification model’s performance and the AUC represents the degree of separability by the classifiers. Therefore, we have distinct performance metrics to display the results from various perspectives.

## 3. Results and Discussion

This section is broken up into numerous subsections that detail the extensive experiments that were carried out for this research and the results of those experiments. The appropriate missing data imputation and feature selection algorithms are studied using comprehensive ablation investigations in Section 3.1 and Section 3.2. Section 3.3 focuses on optimizing various hyperparameters of different ML algorithms. Finally, Section 3.4 concludes by explaining the outcomes obtained from individual ML classifiers as well as our suggested weighted ensemble classifiers with comprehensive ablation analyses. Furthermore, the effectiveness of the proposed classifier was examined by employing a statistical test known as an analysis of variance (ANOVA).

### 3.1. Results for Missing Imputation

To handle the missing data challenge (see Section 2.2.1), we utilized the three most familiar approaches, as stated in Table 4, namely Case Deletion (remove the missing data sample), MEDimpute (using median value), and KNNimpute (utilizing K nearest neighbor data sample). We employed two distinct DDC datasets (DDC-2011 and DDC-2017) and six distinct ML classifiers, namely GNB, BNB, RF, DT, XGB, and LGB, for indirect evaluation [15] in order to determine which MVI technique performs the best when it comes to diabetes classification. Our goal was to determine which MVI technique is the most effective at identifying diabetes cases.

Table 4 demonstrates that the MEDimpute exceeds the Case Deletion and KNNimpute methods in most situations by a substantial margin. The Case Deletion or KNNimpute approach outperforms MEDimpute by a small margin in the remaining circumstances. Particularly for the DDC-2011 dataset, the AUC is significantly higher for RF, DT, XGB, and LGB classifiers, while MEDimpute is employed. Again, the percentage of missing values in the DDC datasets (as described in Section 2.1) is much lower than the total data sample, which is 11.25%. Furthermore, only six features contain missing data out of the thirteen features. However, the missing data numbers and the attributes, including missing data, are relatively minor. Therefore, the resulting AUCs from all of the detection models for all suggested datasets are nearly identical for all MVI approaches, with the MEDimpute method performing significantly better in most cases (see Table 4). Such superior results from the MEDimpute prove its superiority for the MVI in fewer missing values, which is also reviewed in the article [15]. As the MEDimpute strategy beats the other two MVI techniques (see Table 4), this strategy is implemented in the remaining investigation in this research.

### 3.2. FS Results

**Figure 2:**
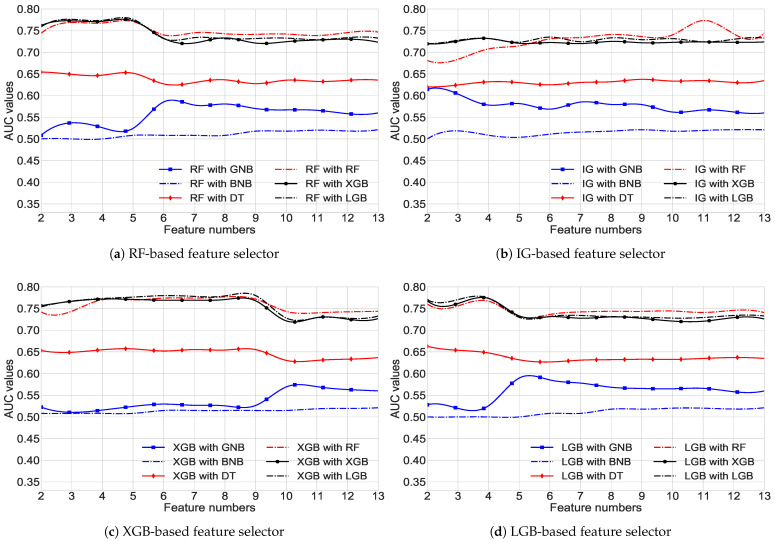
AUC versus feature numbers (2–13) in the submitted DDC dataset, considering four distinct feature-choosing approaches and six different ML-based models.

The proposed methodology now includes FS methods, which were applied to identify the smallest subset of features; as a result, the performance of classifiers has been enhanced. A low level of classification accuracy might be the outcome of using high-dimensional qualities, which can lead to data redundancy or distortion. Therefore, to attain the highest performance, we need to determine the set with the fewest features. Predicting the suitable FS strategy without ablation research is not a viable option due to the fact that the performance of such approaches frequently fluctuates depending on the applications. In order to execute a thorough ablation experiment, this article examines four different FS techniques without feature modification (therefore preserving the interpretation) and six distinct classifiers for the diabetes classification challenge (see results in Table 5 and Figure 2).

The initial stage in FS is to rank features according to importance scores obtained from various algorithms. Table 5 demonstrates the feature importance score according to the four FS methods: RF, IG, XGB, and LGB, utilizing the same dataset and experimental conditions. According to the RF-based FS, the top five features are F13, F5, F11, F12, and F7, whereas the other three FS methods exhibit different features as the top five most significant attributes. Interestingly, F13 (BMI of the respondent) is the supreme feature that is agreed upon by all the FS techniques. In contrast, the other features selected by RF methods also have been selected by other one or two FS strategies. However, further insight discussion for determining the best FS methods has been visualized in Figure 2.

The FS outcomes from various experimental investigations employing four different FS processes are delineated in Figure 2, demonstrating the FS results from different classifiers and exhibiting their related most elevated AUC at the various feature numbers. The findings from RF-based FS techniques corroborate that three classifiers, RF, XGB, and LGB, obtain a loftiest likely AUC of around 0.77 using top 4–5 features (see Figure 2a). The IG-based FS technique, with the highest AUC of 0.77 for the RF model (see Figure 2b), also has the best performance when utilizing the top 11 features. Another XGB-based FS approach shows its highest AUC of 0.78 using the top 8–9 features for the LGB classifier (see Figure 2c). The remaining last one, known as the LGB-based FS method, provides the best AUC of approximately 0.77 for the identical LGB model with the top 2–3 features (see Figure 2d). Although each of the four FS methods determines the different features as their most important attributes (see in Table 5), they do not perform similarly in producing the diabetes classification outcomes, as reflected in Figure 2. As a result, we emphasize the FS model, which can produce improved AUC values for the categorization of diabetes. Again, despite the fact that both RF-based and LGB-based FS techniques obtain the same AUC, the LGB-based FS technique is not employed in this research due to its non-linear and gradually declining performance. As a consequence, the RF-based FS approaches have been regarded as the most essential FS techniques in our pipeline based on the features they have specified. As RF-based FS provides the best possible AUC with the minimum subset of features such as F13, F5, F11, F12, and F7 (higher to lower feature ranking), it is employed in the remaining experiments.

### 3.3. Optimization Results

In order to generate the maximum feasible AUCs using six different ML models, the MVI and FS techniques that yielded the best results during the two earlier investigations are utilized for tweaking hyperparameters. Table 6 elucidates the hyperparameter list for ML prototypes, together with the optimal weights, using the grid search approach that the proposed framework provides. Grid Search Optimization (GSO) is used to determine the optimum hyperparameter values in order to improve the AUC values for the suggested DDC datasets. This experiment was successful in determining the best parameters of those ML models that will be utilized in the upcoming experiments, particularly for the individual ML model and proposed weighted ensemble model evaluation for the same task diabetes classification on the same experimental condition and suggested dataset.

### 3.4. Classifiers’ Results

Table 7 presents the diabetes classification results of a variety of ML models, as well as their ensemble models utilizing the best performing MVI and FS techniques and proposed DDC-2011 and DDC-2017 datasets.

#### 3.4.1. Single ML Model’s Results

Classification of diabetes using the proposed DDC dataset with Bayesian classifiers such as GNB and BNB demonstrates that the BNB model outperforms the GNB model by two cases out of four, with substantial margins for the DDC-2011 dataset. Again, with the other dataset (DDC-2017), the GNB model outperforms the BNB model, which indicates that both Bayesian models are unpredictable and display low accuracy values. For example, the highest was 62.8% for DDC-2011 and 55.6% for DDC-2017 (see Table 7). The fact that the Bayesian classifier assumes that all predictors (attributes) are independent, which is a sporadic occurrence in the real world, causes the targeted study to produce subpar DDC results.

Again, RF and DT tree-based classifiers exhibit that the DT model surpasses the RF model with a significant margin for both the DDC datasets. A close inspection of the RF classifier tells that for the DDC-2011 it is biased toward the positive class (as specificity is 0.0% with 100.0% sensitivity) and for the DDC-2017 towards the negative class (as specificity is 100.0% with 0.0% sensitivity). Again, RF demonstrates unreliable and ambiguous results for two different DDC datasets, while the DT model provides balanced results for both datasets (see Table 7). Although the BNB and RF models yield an Sn of 100.0%, both models should not predict all samples as positive. This is because of a positive predictive value ($P_{r}$) similar to the positive class prior probability ($P_{pos}$) ($P_{r}=\;P_{pos}$). These findings and discussion reveal that using the Bayesian and RF models to classify the dataset with many inter-class homogeneities is not satisfactory for this article’s experimental approval.

Likewise, when the results of the boosting-based classifiers such as XGB and LGB are compared, the XGB has more significant Sn, Acc, and AUC for the DDC-2011 dataset, while the LGB has a better value of Sp. On the other hand, those classifiers for the DDC-2017 dataset expose that LGB has a more satisfactory performance. However, their performances for the DDC dataset and aimed tasks are more promising than the other four tree-based and Bayesian classifiers. The boosting classifiers applied in this article are extreme gradient boosting and one of the well-known gradient boosting procedures (ensemble), which improved interpretation and swiftness in tree-based ML algorithms [31,64]. Additionally, they minimize a regularized (L1 and L2) objective function that integrates a convex loss function and a correction term for model complexity, producing a more generic classification in any given assignment, including the aspired task in this article. In order to achieve more generic results from a particular model for both the DDC datasets, we have designed several variants of weighted ensemble models that are discussed in the following section in an ablation study.

#### 3.4.2. Proposed Ensemble Models’ Results

We have conducted ablation studies to build an appropriate ensemble classifier with improved diabetes categorization results, as it has been revealed that such a classifier yields more profitable results that are experimentally validated in [31,64]. Table 7 displays all the proposed weighted ensemble models’ results, where those suggested ensemble models utilized individual models’ AUC values as a weight.

Two different models of Bayesian, tree-based, and boosting algorithms are combined pair-wise to build three ensemble models such as GNB + BNB, RF + DT, and LGB + XGB, and tested on both the DDC datasets. The results of those classifiers demonstrate better results than the single model working on our DDC dataset independently (see Table 7). Again, the weighted mixture of four different models returns three different ensemble classifiers, namely GNB + BNB + RF + DT, RF + DT + LGB + XGB, and LGB + XGB + GNB + BNB. The obtained results from those three models are enhanced than all previous models for the DDC datasets. The further combination of all the six models to assemble LGB + XGB + GNB + BNB + RF + DT can not produce as good results as the combination of four models.

Furthermore, for the DDC-2017 dataset, the ensemble models with two ML models win three out of four cases such as Sn, Acc, and AUC, by a considerable margin (see 20th–22nd rows of Table 7). Secondly, using the DDC-2011 dataset, the weighted combination of two distinct classifier models, Bayesian with tree-based, Bayesian with boosting-based, and tree with boosting-based, demonstrates that the suggested GNB + BNB + XGB + LGB boosts overall accuracy and Sp while dropping Sn and AUC. However, applying the same aggregation models for the DDC-2017 dataset shows that DT + RF + XGB + LGB enhances the overall accuracy and AUC value. The other two models, GNB + BNB + XGB + LGB and GNB + BNB + DT + RF, can not provide any ensembling success. Ultimately, the weighted ensemble of Bayesian, tree, and boosting-based prototypes does not ameliorate categorization outcomes; instead, it degrades the execution for the DDC-2011 dataset, but for the DDC-2017 dataset improves the overall accuracy.

**Figure 3:**
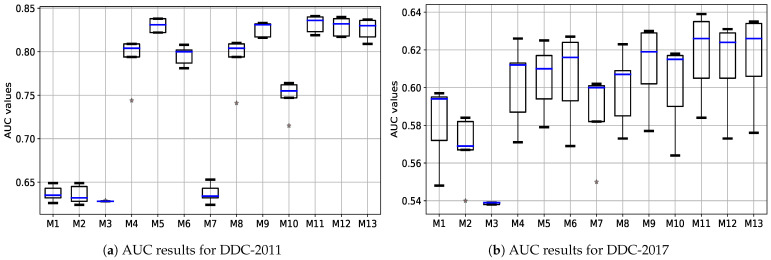
Box and whisker plots of AUC results acquired from 5-fold cross-validation on various ML classifiers, where M-1 to M-13 represent GNB, BNB, RF, DT, XGB, LGB, GNB + BNB, RF + DT, LGB + XGB, GNB + BNB + DT + RF, GNB + BNB + XGB + LGB, DT + RF + LGB + XGB, and GNB + BNB + DT + RF + XGB + LGB, respectively.

Likewise, using a statistical ANOVA test and the 5-fold cross-validation technique, the experimental findings from several classification models employ the proposed best preprocessing method. The AUC results of DDC-2011 and DDC-2017 validation tests are plotted in box and whisker plots in Figure 3a,b, respectively. In ANOVA testing, α=0.05 is considered a threshold for rejecting the void supposition (all models’ mean values are identical) if *p*-value ≤0.05, resulting in significant outcomes. The ANOVA test yields a *p*-value of $3.52\times 10^{-3}\;\left(\leq 0.05\right)$, indicating that an alternate hypothesis is acceptable and none of the mean values are similar (correspondingly depicted in Figure 3). Moreover, the ANOVA test is combined with a post hoc *t*-test to determine the classification model which performs better in the suggested classification scheme, confirming the supremacy of the proposed weighted ensemble DT + RF + XGB + LGB classification model.

#### 3.4.3. Year-Wise Cross-Fold Validation

The previously presented findings have 5-fold cross-validation, and they were achieved by utilizing either the DDC-2011 dataset or the DDC-2017 dataset. In contrast, we recommended using DDC datasets spanning two years (n = 2), with 5-fold cross-validation, and applying three different scenarios to this part. In the first scheme, features are identified by utilizing DDC-2017, and then those selected features are administered into the DDC-2011 dataset, after which the features from both datasets are concatenated with the features that were initially selected. When applied to this synopsis, the feature ranking generates a scale with a higher-to-lower order of F13, F11, F5, F12, and F7, which results in the highest AUC for DDCs. In the second step of the process, features are chosen by referring only to the DDC-2011 dataset. After that, the chosen features are applied to the DDC-2017 dataset, and then those features are concatenated with the features of both datasets. In this particular instance, the sequence of the features used to calculate the optimal AUC is as follows: F13, F5, F11, F12, and F7 (higher to lower order). In the final layout, both datasets are joined together, and the RF approach is then used to select the features of the combined dataset. As a result, the final feature ranking score is F13, F5, F11, F12, and F1, maintaining the higher to lower order. The individual three examples that were employed for feature selection techniques are shown in Table 8 as year-wise cross-validation. Six different ML classifiers and their ensembles are trained and validated using each case separately.

In case-1, when compared to the performance of separate ML models, the XGB classifier achieves much higher results in both Acc and AUC. On the other hand, while looking at the other two situations, it has been seen that LGB performs better in three different variables, namely Sp, Acc, and AUC. Unfortunately, RF displays a sensitivity of 100.0% in all situations; hence, the RF model cannot be considered a reliable model for these DDC datasets. The GNB + BNB+XGB+LGB ensemble classifier achieves a higher Acc and AUC than the individual ML classifiers when applied to case-1 and case-2, respectively. When applied to the case-3 scenario, the DT + RF + XGB + LGB classifier demonstrates superior performance compared to the other ensemble classifiers in terms of Sp, Acc, and AUC.

**Figure 4:**
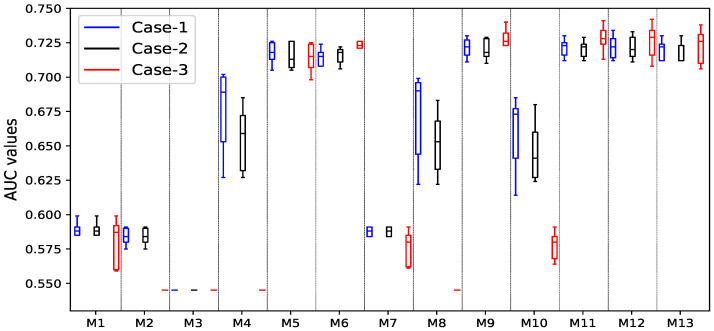
Box and whisker plots of AUC results acquired from 5-fold cross-validation on different ML-based classifiers, where M-1 to M-13 represent GNB, BNB, RF, DT, XGB, LGB, GNB + BNB, RF + DT, LGB + XGB, GNB + BNB + DT + RF, GNB + BNB + XGB + LGB, DT + RF + LGB + XGB, and GNB + BNB + DT + RF + XGB + LGB, respectively.

In addition, a statistical ANOVA test and a 5-fold cross-validation approach are employed in order to evaluate the results of the experiments conducted with the different classification models that made use of the suggested optimal preprocessing method. The results of the validation tests on the consolidated DDC-2011 and DDC-2017 datasets are shown in the form of a box and whisker plot in Figure 4. The ensemble classifier GNB + BNB+XGB+LGB is the top-performing classifier in case-1 and case-2, as shown in Figure 4. On the other hand, for case-3, DT + RF + XGB + LGB is the best performing ensemble classifier.

#### 3.4.4. Comparative Studies

We provide new DDC datasets (see details in Section 2.1), which were used in all of the experiments described in this paper. To the best of our knowledge, utilizing the combined BHDS data of 2011 and 2017–18, there is no work that applied or proposed any ML techniques for early diabetes prediction. This is despite the fact that some studies are attempting to investigate the prevalence of diabetes in Bangladesh as well as the factors that influence the disease [71,72,73,74]. However, according to the findings of research that evaluated ML-based classifiers for automated detection and classification of diabetes in Bangladesh using BHDS 2011 data, the Bagged CART classifier exhibited the greatest area under the ROC curve (AUC) of 0.600 [75]. On the other hand, we employed both BHDS 2011 and BHDS 2017 datasets and were successful in achieving an AUC of 0.832. Using data from the 2011 BDHS, Chowdhury et al. [71] discovered that the overall prevalence of diabetes was 11%, and that the frequency was somewhat lower in males (10.6%) than in women (11.2%). Respondents in the age group of 55–59 years with higher educational achievement and better social status had higher odds of having diabetes than those from a lower age group with no education and lower social status, respectively. They also found that socioeconomic level, location of residence, regions, overweight and obesity, as well as hypertension, were significant correlates with diabetes [71]. Since there are not enough studies that use the same DDC dataset for an accurate comparison, we are unable to compare our findings with those that have been published in a detailed tabular format. As an alternative, we have designed and implemented various variants of ML models and their ensembles.

#### 3.4.5. Strengths and Drawbacks of Our Proposed Ensemble Classifier

Although our predictive ensemble-based model (DT + RF + XGB + LGB) proclaims low accuracy of 73.5%, the results of our article provide a real provocation for the relevant research community to further improve the accuracy rate by using our suggested DDC dataset. However, it offers an acceptable AUC of 83.2%, which is one of the most robust metrics calculated from the ROC curve. The ROC curve represents the true positive rate versus the false positive rate. Therefore, it is evident that the outcomes moderately handle type I and type II errors. One of the constraints of this study is that our algorithm has been applied to only 7529 patients. It would be great to use this algorithm on an enormous population, for example, 10 million people, and check the true positive rate versus the false positive rate. Apart from these limitations, we are now publicly providing our dataset as well as codes so that other researchers could use these as a starting point and propose a new algorithm to predict diabetes and compare it with our results. One of the recommendations is that, as we have applied machine learning and their ensembles, it would be great to explore modern deep learning techniques.

## 4. Conclusions

Employing the suggested ML-based ensemble model, in which preprocessing plays a critical role in ensuring robust and accurate prediction, enabled this research to achieve its goal of making an early prediction of diabetes. The quality of the dataset was improved due to the presented preprocessing technique; the key considerations were selecting features and filling in missing values. The implementation of these preprocessing methods is required, which necessitates doing an exhaustive examination of the ablative processes in order to choose the most suitable approaches. In addition, when compared to previous research, this study produces a more accurate estimation despite including only four to five features, namely the body mass index (BMI) of the respondent, their present age, their average systolic pressure, and their average diastolic pressure, as well as their occupation, which is easily explicable. A weighted ensemble of machine learning classifiers may enhance the categorization consequences according to the suggested framework. This is accomplished by assigning a weight to the probability of the outcomes produced by the ensemble candidates’ models. In terms of its potential to forecast diabetic disease classes in various medical settings, we anticipate that the model that we have developed would display both generality and flexibility. In addition, the extensive DDC dataset that was introduced from the South Asian country of Bangladesh (2011 and 2017–2018), which was the first dataset in this location, will continue to be helpful in future studies that involve the use of demographic information. This dataset can be found at GitHub (https://github.com/kamruleee51/Diabetes-classification-dataset, accessed on 20 September 2022). In addition, the diabetes detection findings of our work provide an open challenge to the associated research community to further improve the results by applying our suggested DDC dataset.

## Figures and Tables

**Table 1:** Overview of different ML-based methods utilized in the previous literature for diabetes prediction, including the year of publication, used dataset, missing value imputation techniques, feature selection strategies, number of selected features, classifier used, and corresponding performance evaluation metrics.

Years	Dataset	MVI ^1^	FS	NSF	BPC	Performance
2016 [32]	ENRC	None	None	9	DT	Acc: 0.840
2018 [37]	LMHC	None	None	All	RF	Acc: 0.808 $S_{n}$: 0.849 $S_{p}$: 0.767
2018 [37]	PIDD	None	mRMR	7	RF	Acc: 0.772 $S_{n}$: 0.746 $S_{p}$: 0.799
2018 [30]	PIDD	None	None	8	NB	AUC: 0.819 Acc: 0.763 $S_{n}$: 0.763
2018 [38]	PIDD	KNN impute	BWA	4	Linear Kernel SVM	AUC: 0.920
2019 [39]	PIDD	NB	None	8	RF	AUC: 0.928 Acc: 0.871 $S_{n}$: 0.857
2019 [40]	PIDD	None	CRB	11	NB	Acc: 0.823
2019 [41]	PIDD	None	None	8	MLP	Acc: 0.775 $S_{n}$: 0.85 $S_{p}$: 0.68
2020 [31]	PIDD	Mean	CRB	6	Ensemble of AB, XGB	AUC: 0.950 $S_{n}$: 0.789 $S_{p}$: 0.789
2020 [42]	NHANES	None	LR	7	RF	AUC: 0.95 Acc: 0.943
2020 [43]	PIDD	Case deletion	None	2	SVM	AUC: 0.700 Acc: 0.750
2021 [44]	PIDD	None	None	8	Ensemble of J48, NBT, RF, Simple CART, RT	AUC: 0.832 Acc: 0.792 $S_{n}$: 0.786
2021 [45]	LMHC	Case deletion	ANOVA, GI	16	XGB	AUC: 0.876 Acc: 0.727 $S_{n}$: 0.738

^1^ Note: MVI: Missing Value Imputation, FS: Feature Selection, NSF: Number of Selected Feature, BPC: Best Performing Classifier, ENRC: Egyptian National Research Center, LMHC: Luzhou Municipal Health Commission, PIDD: PIMA Indian Dataset, mRMR: Minimum Redundancy Maximum Relevance, BWA: Boruta Wrapper Algorithm, CRB: Correlation-Based, NHANES: National Health and Nutrition Examination Survey, ANOVA: Analysis of Variance, GI: Gini Impurity, NBT: Naive Bayes Tree, RT: Random Tree.

**Table 2:** Class label description and class-wise sample distributions of the proposed DDC-2011 and DDC-2017 datasets.

Dataset	Diabetes Patient	Non-Diabetes Patient
DDC-2011	4751	2814
DDC-2017	3492	4073

**Table 3:** The features (categorical/continuous) employed in this research are described in detail. For categorical variables we used an $\chi^{2}$-test, whereas for continuous variables a mean ± std is engaged to represent the substantial relationship with diabetes disease prediction.

Features	Different Features with Short Descriptions	Categorical?	Continuous?	$\chi^{2}$-Test or Mean ± Std	
				DDC-2011	DDC-2017
F1	Division (the respondents’ residence place)	Yes	No	144.689 (0.000)	383.774 (0.000)
F2	Location of respondents’ residence area (urban/rural)	Yes	No	463.00 (0.496)	93.958 (0.000)
F3	Wealth index (respondent’s financial situation)	Yes	No	16.104 (0.003)	482.139 (0.000)
F4	Household’s head sexuality (gender of the household head)	Yes	No	5.858 (0.016)	4.298 (0.117)
F5	Age of household members	No	Yes	54.87 ± 12.94	39.53 ± 16.21
F6	Respondent’s current educational status	Yes	No	6.041 (0.110)	6.960 (0.541)
F7	Occupation type of the respondent	Yes	No	30.430 (0.063)	185.659 (0.000)
F8	Eaten anything	Yes	No	0.663 (0.416)	3.065 (0.216)
F9	Had caffeinated drink	Yes	No	1.590 (0.207)	20.738 (0.000)
F10	Smoked	Yes	No	0.001 (0.985)	7.781 (0.020)
F11	Average of systolic	No	Yes	77.59 ± 12.05	122.63 ± 21.95
F12	Average of diastolic	No	Yes	119.93 ± 21.93	80.52 ± 13.67
F13	Body mass index (BMI) for respondent	No	Yes	2065.63 ± 369.25	2239.43 ± 416.47

**Table 4:** Comprehensive empirical findings for missing value imputation in terms of AUC, utilizing three distinct imputation techniques, two distinct DDC datasets, and six separate ML classifiers. The best imputation strategy has been seen in the blue underline for each dataset and classifier.

Dataset	MVI Techniques	Different ML Classifiers					
		GNB	BNB	RF	DT	XGB	LGB
DDC-2017	Case Deletion	0.597±0.042	0.525±0.020	0.592±0.047	0.518±0.021	0.576±0.063	$\underset{¯}{0.588\pm 0.077}$
	MEDimpute	0.612±0.036	$\underset{¯}{0.526\pm 0.019}$	$\underset{¯}{0.595\pm 0.043}$	0.514±0.022	$\underset{¯}{0.580\pm 0.063}$	0.584±0.080
	KNNimpute	$\underset{¯}{0.616\pm 0.039}$	0.525±0.019	0.589±0.048	$\underset{¯}{0.522\pm 0.019}$	0.576±0.063	0.584±0.078
DDC-2011	Case Deletion	$\underset{¯}{0.577\pm 0.024}$	0.507±0.017	0.493±0.081	0.484±0.031	0.476±0.071	0.485±0.073
	MEDimpute	0.560±0.047	$\underset{¯}{0.521\pm 0.025}$	$\underset{¯}{0.741\pm 0.036}$	$\underset{¯}{0.636\pm 0.017}$	$\underset{¯}{0.727\pm 0.050}$	$\underset{¯}{0.733\pm 0.046}$
	KNNimpute	0.561±0.067	0.517±0.021	0.485±0.074	0.482±0.038	0.476±0.082	0.476±0.082

**Table 5:** Feature importance score in accordance with the four different FS strategies (RF, IG, XGB, and LGB). The five most significant features of individual models are underlined in blue.

FS Methods	Feature Importance Score												
	F1	F2	F3	F4	F5	F6	F7	F8	F9	F10	F11	F12	F13
RF	0.065	0.018	0.048	0.035	$\underset{¯}{0.140}$	0.035	$\underset{¯}{0.072}$	0.018	0.007	0.013	$\underset{¯}{0.125}$	$\underset{¯}{0.116}$	$\underset{¯}{0.308}$
IG	$\underset{¯}{0.006}$	0.00	0.003	0.005	0.00	0.00	0.00	0.00	$\underset{¯}{0.005}$	$\underset{¯}{0.007}$	0.002	$\underset{¯}{0.006}$	$\underset{¯}{0.192}$
XGB	$\underset{¯}{0.073}$	0.034	$\underset{¯}{0.054}$	$\underset{¯}{0.148}$	0.036	0.029	0.035	0.034	0.017	0.039	$\underset{¯}{0.049}$	0.042	$\underset{¯}{0.410}$
LGB	$\underset{¯}{0.074}$	0.024	0.045	0.019	$\underset{¯}{0.143}$	0.03	0.049	0.016	0.007	0.010	$\underset{¯}{0.185}$	$\underset{¯}{0.159}$	$\underset{¯}{0.240}$

**Table 6:** The highest achievable AUC for the DDC dataset with hyperparameters tuning of the six ML models.

Classifiers	Tuned Hyperparameters	AUC (W/ GSO)	AUC (W/O GSO)
GNB	The classes’ prior probabilities (=None) and features’ largest variance portion for stability guesstimate (=0.01).	0.637±0.008	0.628±0.009
BNB	Additive Laplace smoothing parameter (=1.0), classes’ prior probabilities (=None), and to learn or not class priors (=True).	0.637±0.009	0.632±0.003
RF	Bootstrap samples or not (=True), split quality function (=gini), the best split feature numbers (=auto), leaf node number for grow trees (=3), leaf node’s samples (=0.4), the samples required to split an internal node (=2), tree numbers in the forest (=100), out-of-bag samples to calculate the generalization score (=False), and the bootstrapping samples’ randomness control with feature sampling for node’ split (=100).	0.628±0.000	0.628±0.000
DT	Split quality function (=entropy), the best split feature numbers (=auto), leaf node’s samples required (=0.5), samples required to split an internal node (=0.1), the bootstrapping samples’ randomness control with feature sampling for node’ split (=100), and node’s partition strategy (=best).	0.792±0.025	0.675±0.009
XGB	Initial prediction score (=0.5), used booster (gbtree), each levels’ subsample ratio (=1), each nodes’ subsample ratio (=1), evaluation metrics for validation data (=error), minimum loss reduction for a further partition on a leaf node (=1.5), weights’ L2 regularization (=1.5), tree depth (=5), child’s hessian sum (=5), trees in the forest (=100), parallel trees built during each iteration (=1), the bootstrapping samples’ randomness control with feature sampling for node’ split (=100), control the unbalance classes (=1), and training subsample ratio (=1.0).	0.830±0.007	0.811±0.008
LGB	Boosting method (=gbdt), class weight (=True), tree construction’s columns subsample ratio (=1.0), base learner tree depth (=−1), trees in the forest (=50), the bootstrapping samples’ randomness control with feature sampling for node’ split (=100), base learner tree leaves (=25), and training instance subsample ratio (=0.25).	0.796±0.010	0.793±0.012

**Table 7:** Diabetes classification results have been obtained by implementing six individual ML and weighted ensemble models in the proposed DDC-2011 and DDC-2017 datasets, including the imputation of missing value, feature picking, and hyperparameter tuning. The metrics of the best-performing single model are highlighted in bold fonts, whereas the blue underlines are used to indicate them in the proposed ensemble models.

Datasets	Different Classifiers	Sn ↑	Sp ↑	Acc ↑	AUC ↑
DDC-2011	GNB	0.974±0.005	0.037±0.009	0.625±0.003	0.637±0.008
	BNB	1.000±0.000	0.000±0.000	0.628±0.000	0.637±0.009
	RF	1.000±0.000	0.000±0.000	0.628±0.000	0.628±0.000
	DT	0.964±0.048	0.275±0.036	0.707±0.021	0.792±0.025
	XGB	0.937±0.007	0.398±0.022	0.737±0.006	0.830±0.007
	LGB	0.711±0.011	0.662±0.035	0.693±0.013	0.796±0.010
	GNB + BNB	0.974±0.005	0.037±0.009	0.626±0.003	0.637±0.010
	RF + DT	0.988±0.023	0.247±0.010	0.713±0.016	0.791±0.026
	LGB + XGB	0.854±0.009	$\underset{¯}{0.510\pm 0.036}$	0.726±0.008	0.826±0.008
	GNB + BNB + DT + RF	$\underset{¯}{0.989\pm 0.010}$	0.234±0.049	0.708±0.024	0.749±0.018
	GNB + BNB + XGB + LGB	0.959±0.005	0.358±0.012	$\underset{¯}{0.736\pm 0.006}$	0.829±0.010
	DT + RF + XGB + LGB	0.959±0.008	0.357±0.017	0.735±0.008	$\underset{¯}{0.832\pm 0.009}$
	GNB + BNB + DT + RF + XGB + LGB	0.984±0.007	0.316±0.012	0.735±0.007	0.826±0.011
DDC-2017	GNB	0.296±0.115	0.778±0.095	0.556±0.009	0.581±0.019
	BNB	0.264±0.018	0.788±0.012	0.546±0.009	0.569±0.016
	RF	0.000±0.000	1.000±0.000	0.538±0.000	0.538±0.000
	DT	0.358±0.041	0.757±0.058	0.573±0.014	0.602±0.020
	XGB	0.440±0.028	0.705±0.006	0.582±0.012	0.605±0.017
	LGB	0.571±0.030	0.593±0.014	0.583±0.017	0.606±0.022
	GNB + BNB	0.250±0.068	0.809±0.051	0.551±0.010	0.587±0.020
	RF + DT	0.311±0.037	0.801±0.030	0.575±0.014	0.600±0.018
	LGB + XGB	$\underset{¯}{0.490\pm 0.035}$	0.667±0.016	0.585±0.019	0.612±0.020
	GNB + BNB + DT + RF	0.287±0.039	$\underset{¯}{0.819\pm 0.016}$	0.573±0.018	0.601±0.021
	GNB + BNB + XGB + LGB	0.432±0.038	0.712±0.023	0.582±0.015	0.612±0.022
	DT + RF + XGB + LGB	0.418±0.019	0.731±0.016	0.586±0.017	$\underset{¯}{0.618\pm 0.021}$
	GNB + BNB + DT + RF + XGB + LGB	0.401±0.026	0.749±0.016	$\underset{¯}{0.588\pm 0.018}$	0.615±0.022

**Table 8:** Diabetes classification results are shown in case-1, case-2, and case-3, where features are selected from the DDC-2017 dataset, DDC-2011 dataset, and both datasets, including missing value imputation and hyperparameter tuning. The metrics of the best-performing single model are highlighted in bold fonts, whereas the blue underlines are used to indicate them in the proposed ensemble models.

Cases	Different Classifiers	Sn ↑	Sp ↑	Acc ↑	AUC ↑
Merged datasets (Case-1)	GNB	0.904±0.014	0.181±0.016	0.575±0.009	0.587±0.008
	BNB	0.796±0.102	0.288±0.144	0.565±0.010	0.584±0.006
	RF	1.000±0.000	0.000±0.000	0.545±0.000	0.545±0.000
	DT	0.741±0.058	0.484±0.088	0.624±0.014	0.674±0.029
	XGB	0.748±0.013	0.521±0.022	0.644±0.007	0.717±0.008
	LGB	0.641±0.011	0.647±0.010	0.643±0.007	0.715±0.006
	GNB + BNB	0.815±0.070	0.283±0.100	0.573±0.100	0.590±0.007
	RF + DT	0.778±0.040	0.431±0.070	0.620±0.014	0.670±0.031
	LGB + XGB	0.703±0.005	$\underset{¯}{0.585\pm 0.015}$	0.649±0.008	0.721±0.007
	GNB + BNB + DT + RF	$\underset{¯}{0.839\pm 0.026}$	0.340±0.090	0.612±0.028	0.658±0.027
	GNB + BNB + XGB + LGB	0.787±0.006	0.485±0.013	$\underset{¯}{0.650\pm 0.006}$	$\underset{¯}{0.722\pm 0.009}$
	DT + RF + XGB + LGB	0.746±0.016	0.531±0.020	0.649±0.006	0.721±0.006
	GNB + BNB + DT + RF + XGB + LGB	0.803±0.012	0.461±0.019	0.647±0.003	0.720±0.007
Merged datasets (Case-2)	GNB	0.904±0.014	0.181±0.016	0.575±0.009	0.587±0.008
	BNB	0.796±0.102	0.288±0.144	0.565±0.010	0.584±0.006
	RF	1.000±0.000	0.000±0.000	0.545±0.000	0.545±0.000
	DT	0.754±0.023	0.442±0.051	0.612±0.014	0.655±0.022
	XGB	0.734±0.023	0.529±0.027	0.641±0.009	0.715±0.009
	LGB	0.644±0.005	0.650±0.014	0.647±0.006	0.715±0.006
	GNB + BNB	0.815±0.070	0.283±0.100	0.573±0.010	0.590±0.007
	RF + DT	0.835±0.032	0.344±0.053	0.612±0.014	0.652±0.022
	LGB + XGB	0.696±0.014	$\underset{¯}{0.589\pm 0.017}$	0.647±0.009	0.720±0.007
	GNB + BNB + DT + RF	$\underset{¯}{0.891\pm 0.023}$	0.247±0.065	0.598±0.018	0.647±0.021
	GNB + BNB + XGB + LGB	0.783±0.009	0.492±0.014	$\underset{¯}{0.650\pm 0.004}$	$\underset{¯}{0.722\pm 0.008}$
	DT + RF + XGB + LGB	0.748±0.014	0.525±0.018	0.647±0.005	0.721±0.006
	GNB + BNB + DT + RF + XGB + LGB	0.810±0.008	0.456±0.022	0.649±0.006	0.720±0.007
Merged datasets (Case-3)	GNB	0.912±0.014	0.136±0.053	0.559±0.020	0.579±0.017
	BNB	1.000±0.000	0.000±0.000	0.545±0.000	0.545±0.000
	RF	1.000±0.000	0.000±0.000	0.545±0.000	0.545±0.000
	DT	1.000±0.000	0.000±0.000	0.545±0.000	0.545±0.000
	XGB	0.712±0.012	0.560±0.008	0.643±0.007	0.714±0.010
	LGB	0.650±0.010	0.654±0.013	0.652±0.008	0.724±0.010
	GNB + BNB	0.966±0.023	0.043±0.014	0.545±0.008	0.576±0.012
	RF + DT	$\underset{¯}{1.000\pm 0.000}$	0.000±0.000	0.545±0.000	0.545±0.000
	LGB + XGB	0.690±0.009	0.609±0.005	0.653±0.006	0.726±0.010
	GNB + BNB + DT + RF	0.983±0.021	0.016±0.018	0.543±0.003	0.577±0.010
	GNB + BNB + XGB + LGB	0.788±0.018	0.493±0.035	0.653±0.010	0.726±0.012
	DT + RF + XGB + LGB	0.761±0.009	$\underset{¯}{0.530\pm 0.005}$	$\underset{¯}{0.656\pm 0.005}$	$\underset{¯}{0.728\pm 0.009}$
	GNB + BNB + DT + RF + XGB + LGB	0.834±0.017	0.428±0.036	0.649±0.012	0.722±0.012

## Data Availability

This DDC dataset was gathered regarding all ethical points encountered on the DHS websites (https://dhsprogram.com/, accessed on 20 September 2022). This study excluded the ethical consideration acceptance individually. The DDC data and source codes that keep the determinations of this investigation are unrestricted at https://github.com/kamruleee51/Diabetes-classification-dataset, accessed on 20 September 2022.

## Abbreviations

The following abbreviations are used in this manuscript:

ANN	Artificial Neural Network
AB	AdaBoost
Acc	Accuracy
ANOVA	Analysis of Variance
BWA	Boruta Wrapper Algorithm
BPC	Best Performing Classifier
CRB	Correlation-Based
DDC	Diabetes Diseases Classification
DT	Decision Tree
DM	Diabetes Mellitus
FBS	Fasting Blood Sugar
FS	Feature Selection
GI	Gini Impurity
GOSS	Gradient-based One-side Sampling
GSO	Grid Search Optimization
KNN	K-Nearest Neighborhood
KCV	K-fold Cross-Validation
LDA	Linear Discriminant Analysis
LR	Logistic Regression
ML	Machine Learning
MI	Mutual Information
MVI	Missing Value Imputation
mRMR	Minimum Redundancy Maximum Relevance
NHANES	National Health and Nutrition Examination Survey
NSF	Number of Selected Feature
NB	Naive Bayes
PIDD	PIMA Indian Dataset
QDA	Quadratic Discriminant Analysis
RT	Random Tree
RF	Random Forest
SVM	Support Vector Machine
Sn	Sensitivity
Sp	Specificity
WHO	World Health Organization
XGB	XGBoost
